# Functional Profile Evaluation of* Lactobacillus fermentum* TCUESC01: A New Potential Probiotic Strain Isolated during Cocoa Fermentation

**DOI:** 10.1155/2017/5165916

**Published:** 2017-07-20

**Authors:** Tauá Alves Melo, Thalis Ferreira dos Santos, Lennon Ramos Pereira, Hélic Moreira Passos, Rachel Passos Rezende, Carla Cristina Romano

**Affiliations:** ^1^Department of Biological Sciences, State University of Santa Cruz, Ilhéus-Itabuna Road, Km 16 Salobrinho, 45662-900 Ilhéus, BA, Brazil; ^2^Department of Biological Sciences, State University of Feira de Santana, Transnordestina Avenue, S/N, Novo Horizonte, 44036-900 Feira de Santana, BA, Brazil

## Abstract

The use of intestinal probiotic bacteria is very common in the food industry and has been the focus of the majority of research in this field. Yet in recent years, research on extraintestinal microorganisms has greatly increased due to their well-known potential as probiotics. Thus, we studied a strain of* Lactobacillus fermentum* (TCUESC01) extracted from fermenting cocoa. First, we examined the impact of pH on the growth of this strain and studied its survival under conditions similar to those of the human gastrointestinal tract.* L. fermentum *TCUESC01 demonstrated resistance to conditions mimicking the human stomach and intestines and grew well between pH 5 and pH 7. Next, we subjected* L. fermentum *TCUESC01 to storage at 4°C in a milk solution and found that it survived well for 28 days. Lastly, we measured the susceptibility of this strain to numerous antibiotics and its tendency to autoaggregate.* L. fermentum *TCUESC01 showed significant autoaggregation, as well as susceptibility to the majority of antibiotics tested. Overall, our findings support the potential use of this extraintestinal bacterium as a dietary probiotic.

## 1. Introduction

The search for new probiotics is motivated by the knowledge that each strain of microorganisms possesses different properties and could have unique effects on human health. Historically, it was believed that the lactic bacteria in probiotic products had to be sourced from humans due to the specificity of the host [1]. However, extraintestinal microorganisms isolated from fermented lactose-containing foods or fermented vegetables also exhibit promising probiotic effects [2, 3]. Preliminary evidence from our lab indicates that* Lactobacillus* strains derived from the fermentation of high-quality cocoa exhibit probiotic properties: they reduce histological damage, reduce the systemic concentration of inflammatory cytokines, and increase the serum IgA levels in an in vivo experimental model of colitis [4]. However, the possible use of these strains in commercial products depends on a series of tests recommended by international organizations. According to the Food and Agricultural Organization of the United Nations (FAO) and the World Health Organization (WHO), potential probiotic strains should be evaluated for their functional and technological characteristics, including their resistance during gastrointestinal transit and their stability during storage [5]. Therefore, we evaluated the functional properties and safety of the* Lactobacillus fermentum* strain TCUESC01 that was isolated during the fermentation of high-quality cacao.

## 2. Materials and Methods

### 2.1. Microorganisms and Growth Conditions

*Lactobacillus fermentum* TCUESC01 strain (accession number KU244478, GenBank (http://www.ncbi.nlm.nih.gov/nuccore/KU244478)) was cultivated in lactobacilli MRS broth (1% peptone, 0.8% meat extract, 0.4% yeast extract, 2% glycose, 0.5% sodium acetate, 0.2% dipotassium hydrogen phosphate, 0.02% magnesium sulfate heptahydrate, 0.005% manganese sulfate tetrahydrate, and 0.02% citric acid triammonium salt) (HIMEDIA®, India) for 18 h at 37°C and stored at −80°C in a 10% milk solution (Molico®, Brazil) containing 30% glycerol.

### 2.2. Analysis of Growth and Viability under Varied pH

MRS broth solutions of pH 2, pH 3, pH 4, pH 5, pH 6, pH 7, pH 8, and pH 9 were prepared by addition of 1 mol·L^−1^ of hydrochloric acid or 1 mol·L^−1^ of sodium hydroxide. Before the trial,* L. fermentum* TCUESC01 was cultured for 18 h and then diluted in a saline solution (0.85% sodium chloride) to an optical density (OD) of 0.3 as measured at 600 nm (OD_600_ = 0.3). The trials were performed in 96-well microplates (Costar®), wherein 180 *µ*L of MRS at each pH was inoculated with 20 *µ*L of active culture or saline as a control. The microplate was incubated at 37°C and the OD at 600 nm was determined hourly for 10 h using a spectrophotometer (Molecular Devices®, Versamax tunable microplate reader). In parallel, samples were taken every hour from each pH, plated on MRS agar, and incubated under anaerobic conditions at 37°C to test cell viability.

### 2.3. In Vitro Tolerance to Gastrointestinal Conditions

Bacteria were cultured at 37°C overnight in 40 mL of MRS broth, washed in a saline solution, and inoculated into 20 mL of a 10% milk solution. Milk fermentation was allowed to proceed until a pH of 4.5 was reached, at which point the bacteria were counted (CFU·mL^−1^) by serial dilution and plating on MRS agar. In addition, a serial dilution was done in a saline solution (pH 2.5) with pepsin (3 g/L), followed by incubation at 37°C for 1.5 h. Bacteria were washed by two cycles of centrifugation (5000 ×g/10 min) and resuspension in a saline solution, before being resuspended in 20 mL of 1% porcine bile at pH 8.0 (Merck®, Germany) and incubated at 37°C for 45 minutes. The bacterial counts (CFU·mL^−1^) were determined by plating the bacterial solution in MRS agar under anaerobic conditions at 37°C for 48 h after each incubation phase.

### 2.4. Survival during Cold Storage in Acidified Milk

The* L. fermentum* strain TCUESC01 was cultured in MRS broth and then harvested by centrifugation (5000 ×g/10 min). The bacteria were then washed by resuspension in a saline solution and again pelleted by centrifugation. The cultures were inoculated into a sterile solution of 10% nonfat milk that had been acidified to pH 4.5 with lactic acid (Synth®, Brazil). The lactic solution was refrigerated at 4°C and the colony-forming units (CFU·mL^−1^) were counted by serial dilution and plating on MRS agar at 0, 7, 14, 21, and 28 days. The viability of the strain was determined in relation to the zero time point, which was considered to have 100% survival.

### 2.5. Analysis of Autoaggregation

*L. fermentum* TCUESC01 was cultured in 20 mL of MRS broth overnight at 37°C. The bacterial pellet was collected and resuspended in saline solution to an OD of 0.3 at 600 nm (OD_600_ = 0.3). The capacity of* L. fermentum *TCUESC01 for autoaggregation was tested by incubating the suspension in at 37°C and the OD was monitored hourly for 5 h. The percent aggregation (%  *A*) was calculated as follows:

(1) $$\begin{array}{c} \%\;\;A=\left[\;\frac{\left({OD}_{i}-{OD}_{f}\right)}{\left({OD}_{i}\right)}\;\right]\times 100\%, \end{array}$$

where OD_*i*_ is the initial optical density at the zero time point and OD_*f*_ is the optical density at the time of the measurement. The results shown were the averages plus/minus the standard deviations from three experiments.

### 2.6. Antibiotics Susceptibility Testing

*L. fermentum* TCUESC01 was grown for 18 h in MRS broth at 37°C and diluted to 0.5 on the McFarland scale in a saline solution. Antibiotic discs were placed on Mueller-Hinton agar plates that were then inoculated with 100 *µ*L of the active bacteria suspension. The plates were then incubated under anaerobic conditions for 24 h at 37°C. The zones of inhibition around the discs were measured and the bacteria were classified as resistant (*R*), moderately susceptibility (MS), or susceptible (*S*) based on the standards outlined in Table 1. The antibiotic discs used in the susceptibility test were amoxicillin (AMO, LABORCLIN®, Brazil, 10 *µ*g), ciprofloxacin (CIP, LABORCLIN, Brazil, 5 *µ*g), amikacin (AMI, CECON®, Brazil, 30 *µ*g), azithromycin (AZI, CECON, Brazil, 15 *µ*g), amoxicillin and clavulanic acid (AMC, SENSIFAR®, Brazil, 30 *µ*g), norfloxacin (NOR, LABORCLIN, Brazil, 10 *µ*g), sulfonamide (SUL, NEWPROV®, Brazil, 300 *µ*g), vancomycin (VAN, SENSIFAR, Brazil, 30 *µ*g), streptomycin (EST, LABORCLIN, Brazil, 10 *µ*g), erythromycin (ERI, CECON, Brazil, 15 *µ*g), tetracycline (TET, SENSIFAR, Brazil, 30 *µ*g), imipenem (IPM, CECON, Brazil, 10 *µ*g), cefalotin (CFL, LABORCLIN, Brazil, 30 *µ*g), gentamicin (GEN, CECON, Brazil, 10 *µ*g), cefotaxime (CTX, SENSIFAR, Brazil, 30 *µ*g), cotrimoxazole (trimethoprim and sulfamethoxazole) (SUT, SENSIFAR, Brazil, 25 *µ*g), chloramphenicol (CLO, SENSIFAR, Brazil, 30 *µ*g), clindamycin (CLI, CECON, Brazil, 2 *µ*g), penicillin G (PEN10, CECON, Brazil, 10 *µ*g), and cefoxitin (CFO, LABORCLIN, Brazil, 30 *µ*g).

### 2.7. Statistical Analyses

The calculations of means and standard deviations, the analyses of variance, Tukey's Multiple Comparison Tests, and all statistical analyses were done using the GraphPad® Prism 5.0 software program. All graphs were also produced using the GraphPad Prism 5.0 program.

## 3. Results

### 3.1. Effect of pH on* L. fermentum* TCUESC01 Growth and Viability

**Figure 1:**
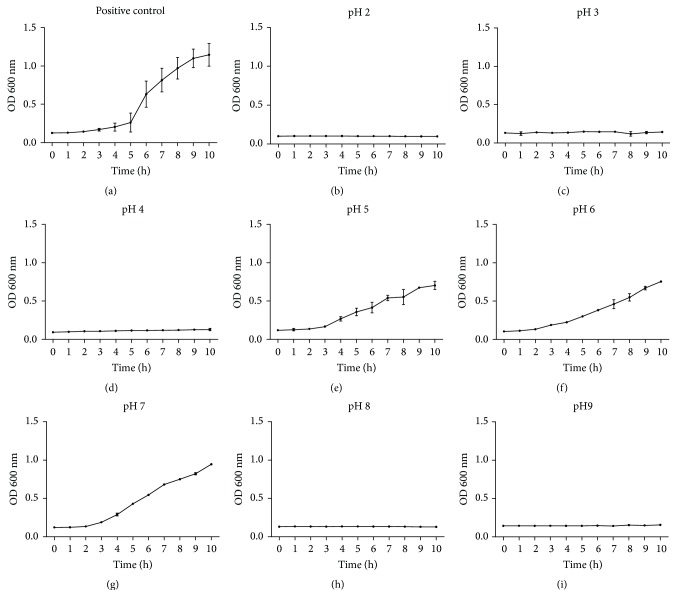
Growth of* Lactobacillus fermentum* TCUESC01 in the period from 0 to 10 hours of cultures at 37°C in different pH: (a) growth in MRS without modification of pH (pH 6.52); (b) growth in MRS with pH 2; (c) growth in MRS with pH 3; (d) growth in MRS with pH 4; (e) growth in MRS with pH 5; (f) growth in MRS with pH 6; (g) growth in MRS with pH 7; (h) growth in MRS with pH 8; (i) growth in MRS with pH 9. Each point of the graphic represents the average and the standard deviation from three experiments.

*L. fermentum* TCUESC01 was able to grow in media at pH 5, pH 6, and pH 7 (Figure 1). However, growth was not observed outside this pH range (Figure 1).

### 3.2. Tolerance of* L. fermentum* TCUESC01 to Gastrointestinal Conditions In Vitro

**Figure 2:**
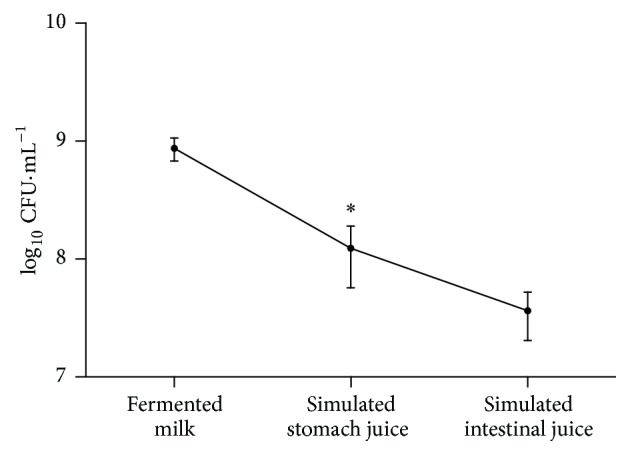
Survival of* Lactobacillus fermentum* TCUESC01 during passage through the simulated gastrointestinal tract. “Fermented milk” after fermentation of the milk; “simulated stomach juice” after passage in saline pH 2.5 + pepsin; “simulated intestinal juice” after passage in ox bile 1%. Each point on the graph represents the average and standard deviant of three experiments.  ^*∗*^Statistically significant reduction (*p* < 0.05) in relation to “fermented milk.”

The tolerance of* L. fermentum TCUESC01* to gastrointestinal passage was evaluated under conditions designed to mimic the human gastrointestinal tract (Figure 2). A bacterial solution was grown to a concentration of 8.7 × 10^8^ CFU·mL^−1^ in a 10% milk solution. After submitting the bacteria to a solution containing pepsin at pH 2.5 for 1.5 h to simulate gastric juice, we observed a statistically significant reduction (*p* < 0.05) of the bacterial concentration to 1.23 × 10^8^ CFU·mL^−1^. After being washed with saline, the bacteria were then subjected to a solution of 1% porcine bile at pH 8.0 for 45 minutes to simulate the intestinal environment. Following this treatment, we observed a reduction of about 1 log in the bacterial count (3.6 × 10^7^ CFU·mL^−1^). The reduction in bacterial counts during incubation in simulated intestinal juice was not statistically insignificant.

### 3.3. Survival of* L. fermentum* TCUESC01 under Commercial Storage Conditions

**Figure 3:**
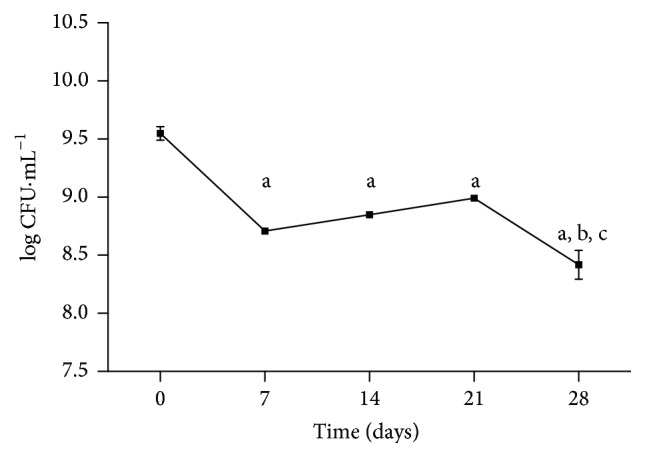
Survival of* Lactobacillus fermentum *TCUESC01 in fermented milk from 0 to 28 days, at 4°C. Each point represents the average and standard deviation of three experiments. “a”: statistically significant difference in relation to day zero (*p* < 0.05); “b”: statistically significant difference in relation to day 14; “c”: statistically significant difference in relation to day 21.

To evaluate their survival during storage,* L. fermentum* bacteria were refrigerated at 4°C for 28 days in an otherwise sterile 10% nonfat milk acidified to pH 4.5 with lactic acid (Figure 3). The bacterial strain was initially at a concentration of 3.6 × 10^9^ CFU·mL^−1^, but after 7 days of storage we observed a statistically significant reduction of approximately 1 log in the bacterial count. From day 7 to day 21, there was unexpected growth from 4.3 × 10^8^ CFU·mL^−1^ to 9.0 × 10^8^ CFU·mL^−1^. By day 28, the bacterial concentration had decreased to 2.83 × 10^8^ CFU·mL^−1^.

### 3.4. Autoaggregation of* L. fermentum* TCUESC01

**Figure 4:**
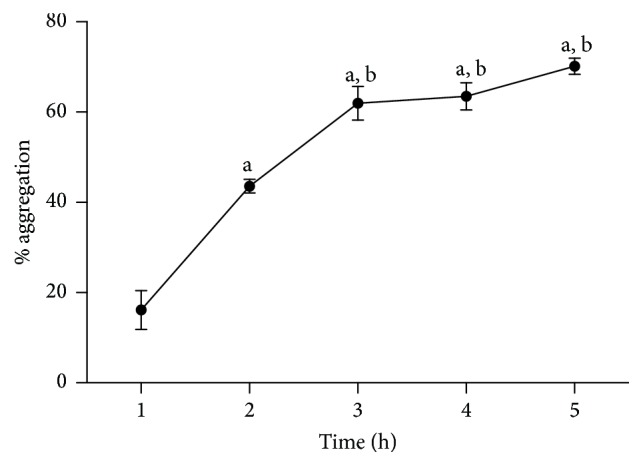
Percentage of autoaggregation of* Lactobacillus fermentum *TCUESC01 evaluated from the 1st to 5th hour of cultivation in MRS broth at 37°C. “a”: statistically significant difference in relation to the 1st hour of aggregation; “b”: statistically significant difference in relation to the 2nd hour of aggregation, *p* < 0.05. Each point represents the average and standard deviation of 3 experiments.

The bacteria increasingly aggregated until the fifth hour of in vitro culture, at which point a maximum of 70.19 ± 1.78% aggregation was observed (Figure 4). However, the hourly increases in the percent aggregation were only statistically significant until the third hour of the experiment (*p* < 0.05).

### 3.5. Susceptibility of* L. fermentum TCUESC01 *to Antibiotics

This strain of* L. fermentum *showed susceptibility to the majority of antibiotics tested (Table 2). The few exceptions were the fluoroquinolones norfloxacin and ciprofloxacin, the nucleic acid synthesis inhibitors sulfonamide and cotrimoxazole (sulfamethoxazole and trimethoprim), the cell wall synthesis inhibiting glycopeptide antibiotic vancomycin, and the cell wall synthesis inhibiting *β*-lactam cefoxitin.* L. fermentum TCUESC01 *was susceptible to amoxicillin, amoxicillin and clavulanic acid, penicillin G, the *β*-lactams cefotaxime and cefalotin, the aminoglycosides amikacin and gentamycin, the lincosamide clindamycin, the carbapenem imipenem, the macrolides azithromycin and erythromycin, the phenicol chloramphenicol, and tetracycline. The strain was also moderately susceptible to streptomycin.

## 4. Discussion

Guidelines established by the FAO and WHO affirm the need to analyze the functional properties and safety of bacteria before proposing its use in a food matrix [5]. We initially evaluated the capacity of this species of* Lactobacillus* to grow and survive at different pH, and although it exhibited growth only in the range from pH 5 to pH 7, it remained viable during 10-h incubations at all pH levels evaluated, with the exception of pH 2. Studies have demonstrated wide variability in the gastric pH when the stomach is empty [9–11], with average values lower than pH 4 [12]. The intestinal environment is more stable and varies between pH 6 and pH 8, depending on the intestinal region evaluated [13, 14]. Therefore, even though this lactic bacterium has not shown the capacity to multiply or survive below pH 2.5, it remains viable in the intestinal pH range and therefore may be able to function in that environment. Consistent with our data,* Lactobacillus plantarum* (ST194BZ, ST414BZ, and ST664BZ),* Lactobacillus rhamnosus* (ST461BZ, ST462BZ), and* Lactobacillus paracasei* (ST242BZ, ST284BZ) isolated from a commonly consumed fermented drink (Boza) from the Balkan Peninsula showed good rates of growth during 10 h of incubation between pH 5 and pH 7 [15].* L plantarum* 423 isolated from sorghum drink,* L. plantarum* 241 isolated from pig ileum,* L. curvatus* DF38 isolated from salami, and* Lactococcus lactis *ssp.* lactis* HV219 isolated from human vaginal secretions also showed growth between pH 5 and pH 6.5 in similar experiments [16]. Overall, our results demonstrate that* L. fermentum* TCUESC01 has growth and pH resistance similar to other potential extraintestinal probiotic bacteria. Furthermore, the sensitivity of the strain to pH levels lower than 2.5 can be overcome by the use of methods that protect the bacteria, such as microencapsulation [17, 18]. Our results support the potential application of this strain as a probiotic additive in foods with distinctly acidic characteristics, for example, cheeses, juices, and fermented milk.

The gastrointestinal environment can be hostile for many bacteria; a variety of stressors such as acidity, digestive enzymes, and biliary salts may negatively influence their survival during transit to the intestine [19]. The* Lactobacillus *in this study showed a discrete quantitative reduction but remained viable under gastric and intestinal conditions and resisted a concentration of bile three times that found in the human intestine (0.3%) [20]. Similar to our data, Kaushik et al. [20] observed that* Lactobacillus plantarum *Lp9 decreased by about 0.5 log from its initial concentration when exposed to conditions that mimic the stomach (pH 2) and 1 log when exposed to conditions that mimic the intestine. In another study,* L. rhamnosus* VT1/1 isolated from cheese showed a reduction approximately 2 log in concentration under low pH conditions (pH 3) and a reduction of 1 log in concentration when incubated at pH 7 in the presence of 2% biliary salts [21]. Our results suggest that* L. fermentum *could move through the gastrointestinal system and survive in concentrations above 10^7^ CFU·g^−1^ (or CFU·mL^−1^), which previous studies suggest would be sufficient to interact and/or interfere with the host environment [22–24].

The food matrix is also an influencing factor in the viability of microorganisms during their storage [18, 25]. In testing the long-term survival of* L. fermentum *TCUESC01 in acidified milk, we observed an initial reduction of the bacterial counts followed by a slight increase from day 7 to day 21. This growth can be explained by continued bacterial metabolism in the lactic solution, although at a reduced rate due to the low temperature. Donkor et al. [26] also observed quantitative variation in probiotic bacteria during storage at 4°C, especially* Lactobacillus delbrueckii *ssp.* bulgaricus *Lb1466 that exhibited growth of 1 log from day 7 to day 14 of cold storage. In another study,* L. plantarum* stored in fermented milk significantly reduced its cellular concentration by 1 log during approximately 28 days of storage at 4°C [27]. Although* L. fermentum* had exhibited a decrease of 1 log from its initial concentration by the last day of storage, its concentration was above average on the expiration date of the lactic solution [28]. Similarly, based on the recommendations of the National Agency for Sanitary Monitoring (ANVISA),* L. fermentum *TCUESC01 could be introduced into food matrices similar to fermented milk and survive in adequate concentrations until the expiration date of the product [29].

Microorganisms with the ability to autoaggregate remain in the intestines for a longer time and thus have better interactions with epithelial cells and the host immune system [30]. The* L. fermentum *TCUESC01 strain demonstrated an elevated capacity for autoaggregation in our 5-h trial. This result is higher than that reported by Beganović et al. [31], who demonstrated that* L. fermentum* A8 had 60.9 ± 3.91% autoaggregation after 5 h of incubation, or that reported by Bao et al. [32], who demonstrated autoaggregation of less than 28% for 10 strains of* L. fermentum *after a 20-h incubation. Based on our results,* L. fermentum* aggregates well and if ingested would likely be able to persist in the human intestinal environment for long time periods.

Finally, we evaluated susceptibility of TCUESC01 to a variety of antibiotics. Knowledge of antibiotic susceptibility is extremely important when we consider three important factors: the rare possibility of infection by* Lactobacillus,* the risk of horizontal transfer of resistance genes to native microbes, and the association between probiotic bacteria and antibiotic treatment.* L. fermentum *TCUESC01 exhibited susceptibility to the majority of the antibiotics, with the exception of nucleic acid synthesis inhibitors (norfloxacin, ciprofloxacin, sulfonamide, and cotrimoxazole) and two cell wall synthesis inhibitors (vancomycin and cefoxin). These results corroborate data published by Kirtzalidou et al. [33] on 74 strains of* Lactobacillus *ssp. isolated from human feces, of which 94.5% strains were resistant to amikacin, all were resistant to kanamycin and ciprofloxacin, 84.7% of strains were resistant to vancomycin, 1.6% strains were resistant to cefalotin, and 8.5% of strains were resistant to bacitracin. In general, lactobacilli show intrinsic resistance to quinolones, trimethoprim, sulfonamides, vancomycin, and the majority of nucleic acid inhibitors, while showing susceptibility to protein synthesis inhibitors with the exception of aminoglycosides [34–38]. It is worth noting that the resistance to antibiotics observed here is intrinsic to the genus as evident from published studies, and horizontal gene transfer is therefore uncommon. In summary, the resistance profile of* L. fermentum *TCUESC01 supports the possibility of use together with antibiotics that work by inhibiting nucleic acid synthesis.

## 5. Conclusions

Despite being an extraintestinal strain isolated during cocoa fermentation,* L. fermentum* TCUESC01 shows strong potential as a probiotic for application in food products. It remains viable across a wide pH spectrum and is therefore suitable for inclusion in different types of foods. When stored in a refrigerated milk product, it maintains viability above the levels recommended by recognized national and international organizations until the product expiration date. Under conditions that mimic gastrointestinal transit, it also survives in quantities sufficient for the maintenance of probiotic potential. In terms of its predicted behaviors within the intestines,* L. fermentum* TCUESC01 shows a strong tendency to autoaggregate. Finally, this strain exhibits antibiotic susceptibility and resistance profiles that will allow for its use alongside drug therapies. Taken together, these characteristics suggest* L. fermentum *TCUESC01 has great potential as a safe probiotic food additive.

## Figures and Tables

**Table 1:** Standards for interpreting the zones of inhibition for specific antibiotics.

Antibiotic	Amount on disc *µ*g	Zone of inhibition (mm)^*∗*^		
		*R*	MS	*S*
Amoxicillin and clavulanic acid	30	≤18	19-20	≥21
Amikacin	30	≤15	16-17	≥18
Amoxicillin	10	≤13	14–16	≥17
Azithromycin	15	≤2	4	≥8
Cefalotin	30	≤14	15–17	≥18
Cefotaxime	30	≤14	15–22	≥23
Cefoxitin	30	≤14	15–17	≥18
Ciprofloxacin	5	≤13	14–18	≥19
Clindamycin	2	≤8	9–11	≥12
Chloramphenicol	30	≤13	14–17	≥18
Cotrimoxazole	25	≤10	11–15	≥16
Erythromycin	15	≤13	14–17	≥18
Streptomycin	10	≤11	12–14	≥15
Gentamicin	10	≤12	—	≥13
Imipenem	10	≤13	14-15	≥16
Norfloxacin	10	≤13	14–18	≥19
Penicillin G	10	≤19	20–27	≥28
Sulfonamides	300	≤12	13–16	≥17
Tetracycline	30	≤14	15–18	≥19
Vancomycin	30	≤14	15-16	≥17

^*∗*^Ranges of zone of inhibition diameters exhibited by bacteria considered susceptible (*S*), moderately susceptible (MS), or resistant (*R*) to each antibiotic are shown [6–8].

**Table 2:** Susceptibility of *L. fermentum* TCUESC01 to antibiotics.

Antibiotic	Zone of inhibition (mm)^*∗*^	Characterization^*∗∗*^
Amikacin	19	*S*
Amoxicillin	47	*S*
Amoxicillin and clavulanic acid	43	*S*
Azithromycin	30	*S*
Cefalotin	23	*S*
Cefotaxime	35	*S*
Cefoxitin	12	*R*
Ciprofloxacin	0	*R*
Clindamycin	14	*S*
Chloramphenicol	30	*S*
Cotrimoxazole	0	*R*
Erythromycin	33	*S*
Streptomycin	13	MS
Gentamicin	15	*S*
Imipenem	57	*S*
Norfloxacin	0	*R*
Penicillin G	30	*S*
Sulfonamides	0	*R*
Tetracycline	20	*S*
Vancomycin	0	*R*

^*∗*^Diameters are shown. ^*∗∗*^Based on standards shown in Table 1, *L. fermentum *TCUES01 is characterized as either susceptible (*S*), moderately susceptible (MS), or resistant (*R*) to each antibiotic tested.
